# Therapeutic Guidelines for the Self-Management of Major Depressive Disorder: Scoping Review

**DOI:** 10.2196/63959

**Published:** 2025-03-06

**Authors:** Priscila de Campos Tibúrcio, Priscila Maria Marcheti, Daniela Miori Pascon, Marco Antônio Montebello Junior, Maria Alzete de Lima, Carla Sílvia Fernandes, Célia Samarina Vilaça de Brito Santos, Maria do Perpétuo Socorro de Sousa Nóbrega

**Affiliations:** 1 School of Nursing University of São Paulo São Paulo Brazil; 2 School of Nursing Federal University of Mato Grosso do Sul Campo Grande Brazil; 3 School of Nursing Pontifical Catholic University of São Paulo Sorocaba Brazil; 4 Sorocaba Engineering Department FACENS University Center Sorocaba Brazil; 5 School of Nursing Federal University of Rio Grande do Norte Rio Grande do Norte, Natal Brazil; 6 Nursing Department Porto School of Nursing Porto Portugal

**Keywords:** major depressive disorder, nursing, revision, self-management, symptoms, PRISMA

## Abstract

**Background:**

Major depressive disorder contributes to the global burden of mental illness. Therapeutic guidelines promote treatment self-management and support caregivers and family members in this process.

**Objective:**

We aimed to identify therapeutic guidelines for the symptoms of major depressive disorder.

**Methods:**

This scoping review followed the assumptions established by the Joanna Briggs Institute and the PRISMA-ScR (Preferred Reporting Items for Systematic reviews and Meta-Analyses extension for Scoping Reviews) protocol, carried out in 12 databases (LILACS, PubMed, SciELO, Scopus, Web of Science, b-on, BDENF, AgeLine, Cochrane, BVS, IBECS, and CINAHL) and 5 secondary gray literature sources (Google Scholar, Global ETD Search, EBSCO Open Dissertations, CAPES Catalog of Theses and Dissertations, and the Digital Library of Theses and Dissertations of the University of Sao Paulo). The eligibility criteria were based on the population, concept, and context framework: people diagnosed with major depressive disorder aged >18 years (population), therapeutic guidelines for self-management of major depressive disorder symptoms (concept), and symptoms of major depressive disorder (context). Data collection was carried out from March to July 2022 and updated in June 2024. The included studies were experimental, quasi-experimental, analytical observational, descriptive observational, qualitative, or quantitative studies; systematic reviews and meta-analyses; and scoping and literature reviews published in full without time restrictions in English, Spanish, or Portuguese. All the information, as well as the studies captured, was stored in a Microsoft Excel spreadsheet using Rayyan and the *JBI Manual for Evidence Synthesis*. The titles, abstracts, and full texts were carefully read and classified, extracting the results. After review by 2 independent researchers, 62 studies were selected. The results are presented descriptively, including characterization of the studies and mapping and categorization of groups and subgroups of therapeutic guidelines for self-management of major depressive disorder.

**Results:**

In total, 62 studies published between 2011 and 2023 were included, where 44 (71%) came from indexed data sources and 18 (29%) were gray literature indexed on Google Scholar (13/62, 21%), doctoral theses (3/62, 5%), and master’s dissertations (2/62, 3%). Among the therapeutic guidelines identified, mapped, and categorized, 7 major groups were identified for self-management: psychotherapy (32/62, 52%), adoption of healthy habits (25/62, 40%), integrative and complementary practices (17/62, 27%), relaxation techniques (9/62, 14%), consultation with a health professional (14/62, 22%), pharmacological therapy (9/62, 14%), and leisure or pleasurable activities (4/62, 6%).

**Conclusions:**

It was possible to identify therapeutic guidelines to promote self-management of major depressive disorder in the adult population. Therapeutic guidance is an important resource for patients, their families, and the community, making patients the protagonists of their own health. For health professionals, therapeutic guidelines become tools that help develop skills and competencies for care among patients, thus ensuring their ability to self-manage major depressive disorder.

## Introduction

### Background

Major depressive disorder is one of the most prevalent mental disorders in the world, as well as one of the clinical conditions that contributes most to the global burden of mental illnesses [1]. The number of people presenting with symptoms suggestive of this disorder has shown a significant increase at an alarming and worrying rate, and it is estimated that it will be the world’s first major disabling public health problem by 2030 [2].

It is a classic mental disorder that involves evident alterations in affect, cognition, and neurovegetative functions, affecting approximately 300 million people worldwide [2]. It is estimated that 3.8% of cases lead to functional incapacity and damage to physical and mental health, as well as professional losses and considerable morbidity and mortality due to suicide or association with other illnesses [3].

In addition to these impacts, it is difficult to diagnose major depressive disorder accurately and quickly as its classification and assessment are based on clinical findings and the patient’s history [2]. Therapies for the treatment of major depressive disorder, such as psychobiosocial therapies, psychotherapy, and pharmacotherapy, have advanced and are used according to the severity of the symptoms and potential adverse events [4]. Considering that mental health care has undergone important transformations, such as the creation of an out-of-hospital network made up of substitute services that propose the rescue of the singularity or subjectivity of the person in psychological distress, respecting their integrality and existence [5], promoting their autonomy in the management of the illness or treatment means encouraging empowerment, the ability to make choices, and taking responsibility for themselves as a citizen and social being [6].

This knowledge and skills are important tools to prevent complications, control symptoms, self-manage treatment, and even avoid recurrences and hospitalizations [7]. Due to the complexity of the disease, some adaptive actions can be taken, including controlling destabilizing factors, preserving self-confidence, re-establishing important relationships, attempting to regain functioning, managing symptoms, negotiating the care environment, and maintaining satisfying relationships [7,8].

Society has been moving toward the construction and adoption of technological innovations using smartphones and computer applications. These resources can be of great value in the process of promoting people’s skills in the mental health field [8]. In this sense, based on the results of this scoping review, a systematic set of therapeutic guidelines was developed based on clinical practice and scientific evidence. This set of therapeutic guidelines is capable of supporting a computational application, generating knowledge in the area of mental health, creating a connection with the patient, and supporting self-management of one’s health remotely [8,9].

These therapeutic guidelines can be adopted with the aim of promoting and supporting self-management in the daily treatment of the patient and supporting the caregiver or family members in performing techniques safely and appropriately, completing therapeutic sessions, maintaining functionality, or preventing possible complications of the disorder [9]. It should be emphasized that, although it is important to encourage self-management by the individual and the family, it is also essential that the care provided by health professionals, including nurses, assists in the promotion and reorganization of the self, breaks and overcomes relationships of dependence, and levels the acquisition or development of knowledge and skills for self-management [7,9].

Given the extent of the symptomatology and the particularities of major depressive disorder, a scoping review allows for an exhaustive analysis of the studies available so that the patient has access to information that will allow them to develop attitudes, knowledge, and skills to self-manage based on the best scientific evidence.

### Objectives

Similarly, a set of therapeutic guidelines will constitute an instrument for the clinical practice of nurses and of the multidisciplinary team as the deepening of specific skills will allow for the provision of individualized care that is more suited to the needs of the person with major depressive disorder. Therefore, this scoping review was carried out with the aim of identifying therapeutic guidelines in scientific production for the self-management of major depressive disorder symptoms.

## Methods

### Overview

A scoping review is used to map key concepts, examine existing evidence before conducting a systematic review, and clarify and define conceptual boundaries. This scoping review was prepared in accordance with the methodological proposal of the Joanna Briggs Institute (JBI) in 2020, supported by the PRISMA (Preferred Reporting Items for Systematic reviews and Meta-Analyses) [10] and PRISMA extension for Scoping Reviews (PRISMA-ScR) [11] guidelines based on the following steps: formulation of the research question, identification of relevant studies, selection of studies, extraction and analysis of data, and synthesis and construction of the report. The PRISMA-ScR checklist is available in Multimedia Appendix 1. This review protocol was registered in the Open Science Framework platform [12] on November 11, 2023, and was modified on February 19, 2025. There was no deviation from the protocol registered on the Open Science Framework platform.

### Research Question

To formulate the guiding research question and guide data collection, the population, concept, and context (PCC) framework was used. The PCC strategy was adopted to drive the research question of this scoping review. In this study, *population* refers to people diagnosed with major depressive disorder aged >18 years, *concept* refers to therapeutic guidelines for self-management of major depressive disorder symptoms, and *context* refers to major depressive disorder symptoms (eg, changes in weight, appetite, and sleep; feelings of guilt; irritability or bad mood; anhedonia; fatigue; and low self-esteem).

Therefore, the following guiding question was defined: “What therapeutic guidelines support self-management of changes in weight, appetite, sleep, feelings of guilt, irritability or bad mood, anhedonia, fatigue and low self-esteem?”

### Study Design and Eligibility Criteria

The data search was carried out in 12 information sources (LILACS, PubMed, SciELO, Scopus, Web of Science, b-on, BDENF, AgeLine, Cochrane, BVS, IBECS, and CINAHL) and 5 secondary information sources of gray literature (Google Scholar, Global ETD Search, EBSCO Open Dissertations, CAPES Catalog of Theses and Dissertations, and the Digital Library of Theses and Dissertations of the University of Sao Paulo).

The search strategy was developed using the controlled and noncontrolled descriptors obtained in the initial search plus the Boolean operators “OR,” “NOT,” and “AND,” as well as keywords found in the Health Sciences Descriptors and Medical Subject Headings of the US National Library of Medicine combined with each other according to each database. The strategies used and how they were combined are available in Multimedia Appendix 2.

The search followed five distinct phases according to the JBI methodology, and a team of 5 researchers was assembled for this scoping review: (1) initial search in the selected databases to identify articles on the topic and, from there, select words and indexing terms contained in these publications to develop the full search strategy; (2) use of the keywords and indexing terms identified to search all the databases included; (3) definition of the study design and eligibility criteria, including the determination of the type of study, target population, interventions and outcomes analyzed. In addition, inclusion and exclusion criteria were established for the selection of studies; (4) identification and selection of studies, including the systematic search in databases and sources of gray literature, application of filters and search strategies to ensure the identification of relevant studies and screening of relevant articles (reading of titles, abstracts and, when necessary, the full text); and (5) data extraction and analysis, including the collection of information from the selected studies, the assessment of methodological quality and the synthesis of findings.

The eligibility criteria are described in Textbox 1, and the studies found were refined based on the PCC acronym, type of study, year of publication, language, and results. The search was carried out from March 2022 to July 2022 and updated in June 2024.

**Eligibility criteria.**

**Inclusion criteria**

- Population: people diagnosed with major depressive disorder aged >18 years
- Concept: therapeutic guidelines for the self-management of symptoms of major depressive disorder
- Context: symptoms of major depressive disorder, such as changes in weight, appetite, and sleep; feelings of guilt; irritability or bad mood; anhedonia; fatigue; and low self-esteem
- Type of study: experimental and quasi-experimental studies (randomized and nonrandomized controlled trials), analytical observational studies (prospective and retrospective cohort studies, case-control studies, and analytical cross-sectional studies), descriptive observational studies (case series, individual case reports, and descriptive cross-sectional studies), qualitative and quantitative studies, systematic reviews, meta-analyses, scoping reviews, and literature reviews; due to the scarcity of publications found in the databases, it was also decided to include gray literature (course completion studies, dissertations, and theses)
- Year: there were no restrictions on the search period as the first search strategies developed yielded few studies on the subject
- Language: English, Spanish, and Portuguese
- Results: publications whose objective addressed therapeutic guidelines to assist in the self-management of patients with active symptoms of major depressive disorder

**Exclusion criteria**

- Population, concept, and context: publications without information on the population, concept, and context of interest for this study
- Type of study: duplicate studies, opinion articles, letters to the editor, abstracts of conference proceedings, and studies with unavailable text
- Language: studies in languages other than those selected
- Results: publications that did not fit the objectives and did not contain information related to the population, concept, and context of this study

### Study Identification and Selection

The process of identifying and selecting studies is one of the most important stages in the conduct of a scoping review. To minimize potential bias, the *JBI Manual for Evidence Synthesis* guidelines were used and explained in a meeting with 6 researchers to ensure that this process was clear to everyone.

Thus, to maintain the rigor of the screening process, the studies obtained from each of the databases based on the eligibility criteria were exported to a reference management software (EndNote; Clarivate Analytics), and duplicates were removed. The remaining studies were then imported into the Rayyan application (Qatar Computing Research Institute) for analysis and selection based on titles and abstracts.

The titles and abstracts were read and analyzed by 2 independent reviewers to identify those that were potentially eligible. After reading the titles and abstracts, the preselected studies were subjected to data analysis and mapping, which consisted of carefully reading and classifying the texts from which the results were extracted.

The selected studies were read in full by 2 additional reviewers (MdPSdSN and PdCT) to confirm their relevance to the research question. Any doubts about the inclusion of studies would be resolved through discussion with a third reviewer until a consensus was reached. It was not necessary to contact a third reviewer.

### Data Extraction and Analysis

As recommended by the JBI, the authors developed a tool to extract the data for the scoping review. Those responsible for data extraction (MdPSdSN, PdCT, and PMM) read and carefully classified the texts. The data were documented in a Microsoft Excel (Microsoft Corp) spreadsheet for better data extraction and evaluation. The final studies were determined from this step. The following variables were extracted from the studies: symptoms, authors, year, reference, study title, objective, study design, and mapping of results.

The focuses of the studies were analyzed, and the results and discussion are presented descriptively and quantified as frequencies in tables. The Discussion section synthesizes the evidence found during the review to explore it and compare it with the existing literature.

The mapped results were categorized into 7 major groups and subgroups of therapeutic orientations for the self-management of major depressive disorder based on the similarities between them. For example, therapeutic orientations focused on physical activity or regular exercise and healthy eating were classified as adoption of healthy habits, whereas listening to music, watching movies, reading, painting, going out with friends, taking outdoor walks, and practicing spiritual and religious activities were grouped in the category of leisure or pleasurable activities. This was done for all the therapeutic orientations identified considering the objective of this scoping review.

Regarding the methodological assessment (although it was not mandatory), each included study had its level of evidence identified based on the JBI appraisal tools [13] and the study design.

Thus, classification I was assigned to systematic reviews and meta-analyses of randomized clinical trials, classification II was assigned to randomized clinical trials, classification III was assigned to nonrandomized controlled trials, classification IV was assigned to case-control or cohort studies, classification V was assigned to systematic reviews of qualitative or descriptive studies, classification VI was assigned to qualitative or descriptive studies, and classification VII was assigned to opinions of authorities or expert committee reports. This hierarchy classifies levels I and II as strong, levels III to V as moderate, and levels VI to VII as weak.

## Results

### Overview of the Studies Found

Initially, 258,509 potentially eligible publications were found in the databases and 13,081 publications in the gray literature sources, of which 1538 (0.63%) focused on sleep disorders, 8315 (3.39%) focused on appetite disorders, 6732 (2.74%) focused on weight disorders, 5814 (2.37%) focused on feelings of guilt, 2509 (1.02%) focused on irritable mood, 200,002 (81.46%) focused on low self-esteem, 1848 (0.75%) focused on anhedonia, and 31,832 (7.64%) focused on fatigue.

The inclusion and exclusion criteria were applied, and 247,348 duplicate studies were removed, leaving 11,242 (4.55%). Next, a new screening was carried out in search of available publications that had abstracts and dealt with therapeutic guidelines. Of the remaining 11,242 studies, 10,000 (88.95%) were excluded, leaving 1242 (11.05%).

**Figure 1:**
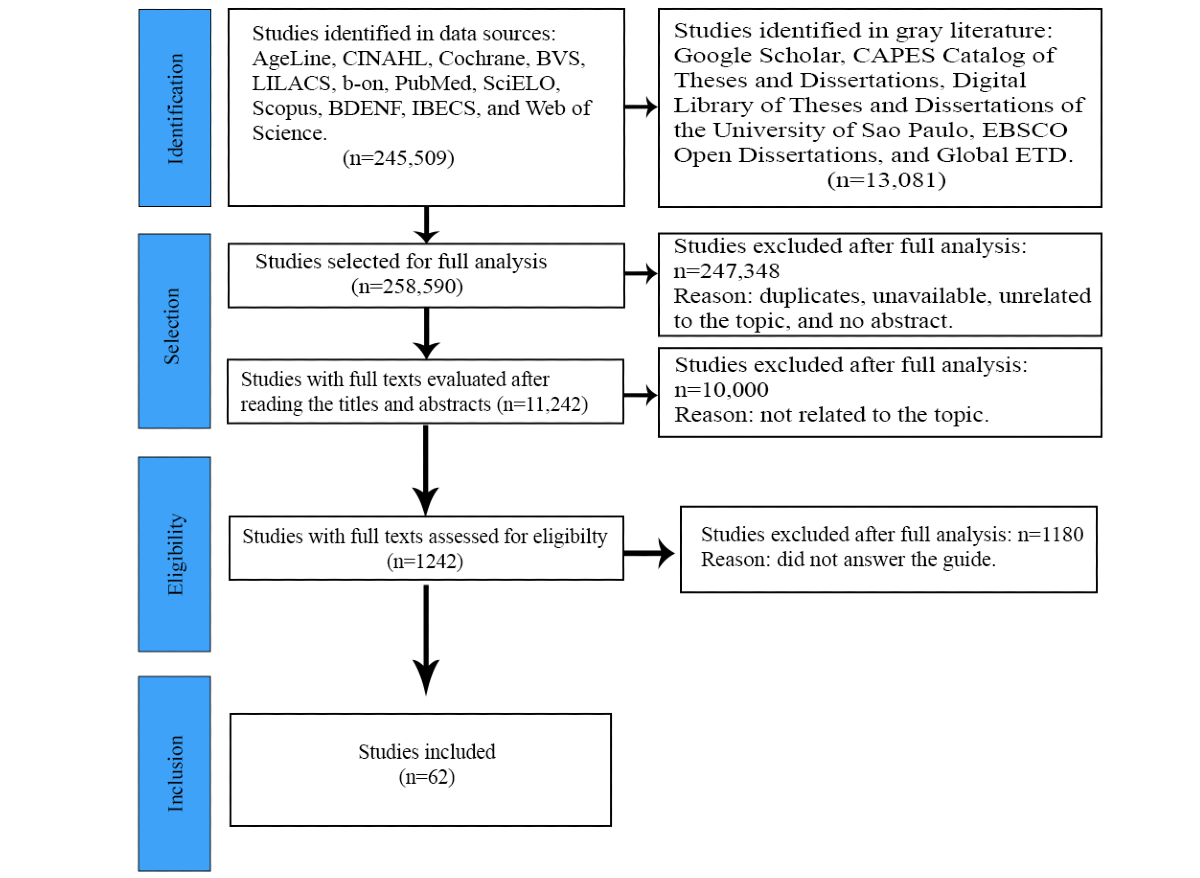
Flow diagram of the search and selection of studies according to the PRISMA (Preferred Reporting Items for Systematic Reviews and Meta-Analyses) 2020 model.

Once the 1242 studies had been selected, the second full reading was carried out. Of the 1242 studies, 1180 (95.01%) were excluded because they did not answer the guiding question. Thus, 62 studies were included in the final sample of this review. Figure 1 shows the results using the PRISMA-ScR flow diagram.

### Characteristics of the Included Studies

Of the 62 studies dealing with therapeutic guidelines (Table 1), 71% (n=44) came from indexed data sources, and 29% (n=18) were gray literature indexed on Google Scholar (n=13, 21%), doctoral theses (n=3, 5%), and Master’s dissertations (n=2, 3%).

It was found that the studies were published between 2011 and 2023, with a higher number of publications in the years 2018 to 2023 (40/62, 63%), demonstrating that the use of therapeutic guidelines is recently evolving. In terms of language, most publications were in English (North America—United States) and Portuguese (South America—Brazil), totaling 63% (40/62) of the publications (Table 1).

Regarding study design, the studies were clinical trials (30/62, 48%), systematic reviews (6/62, 10%), narrative literature reviews (7/62, 11%), systematic reviews and meta-analyses (6/62, 10%), qualitative studies (3/62, 5%), pilot studies (3/62, 5%), experimental studies (2/62, 3%), case reports (2/62, 3%), noncontrolled studies (1/62, 2%), cross-sectional studies (1/62, 2%), and scoping reviews (1/62, 2%; Table 1).

**Table 1:** General characterization of the included studies (N=62).

Category and variable		Studies, n (%)
**Year of publication**		
	2011-2017	22 (37)
	2018-2023	40 (63)
**Origin of the studies**		
	America	40 (63)
	Europe	10 (17)
	Asia	9 (15)
	Oceania	2 (3)
	Africa	1 (2)
**Study design**		
	Clinical trial or experimental, pilot, qualitative, or quantitative study	38 (61)
	Systematic review and meta-analysis, scoping review, or literature review	20 (32)
	Noncontrolled design, case study, or cross-sectional study	4 (7)

### Summary of the Extracted Variables

Table 2 presents the variables extracted from the studies: symptoms, authors, year of publication, reference, title of the study, objective, and study design. All 62 studies focused on people aged >18 years with a diagnosis of major depressive disorder and active symptoms, with a maximum age of 70 years. Of the 15 publications selected on the symptom of sleep disorders, 10 (67%) were published on PubMed [14-23], 1 (7%) was published on SciELO [24], 1 (7%) was published on AgeLine [25], and 3 (20%) were published as gray literature [26-28].

**Table 2:** Studies found according to symptom, study title, objective and study design.

Symptom and study	Study title	Objective	Study design
Sleep disorders; Yang [26], 2019	“Chinese medicine for major depressive disorder: clinical evidence, patients’ experience and expectations, and doctors’ perceptions”	To evaluate and understand the evidence on Chinese medicine for major depressive disorder in the literature	Randomized clinical trial; level II
Sleep disorders; Geoffroy et al [14], 2018	“Insomnia and hypersomnia in major depressive episode: prevalence, sociodemographic characteristics and psychiatric comorbidity in a population-based study”	Examine the frequency of sleep complaints, co-occurrences, sociodemographic characteristics, and psychiatric comorbidities associated with each type of sleep profile	Randomized clinical trial; level II
Sleep disorders; Leggett et al [15], 2018	“Bright light as a preventive intervention for depression in late-life: a pilot study on feasibility acceptability and symptom improvement”	Examine the feasibility and acceptability of a portable bright light and its impact on sleep disorders and symptoms of depressive disorders in older adults	Pilot study; level II
Sleep disorders; Luca et al [16], 2013	“Sleep disorders and depression: brief review of the literature, case report, and nonpharmacologic interventions for depression”	Review and discussion of sleep disorders during major depressive disorder (in particular night terrors, nightmares, hypersomnia, and insomnia)	Case report; level VI
Fatigue; Hulme et al [29], 2018	“Fatigue interventions in long term, physical health conditions: a scoping review of systematic reviews”	Map effective guidelines in all clinical conditions for the symptom of fatigue and whether these guidelines can be applied to other symptoms	Scoping review; level I
Sleep disorders; de Sousa Ibiapina et al [24], 2022	“Effects of music therapy on symptoms of anxiety and depression in adults diagnosed with mental disorders: a systematic review”	Identify and synthesize the evidence from studies that have evaluated the effects of music therapy on the symptoms of anxiety and depression in adults with mental disorders	Systematic literature review; level I
Fatigue; Macnamara et al [30], 2018	“Personalized relaxation practice to improve sleep and functioning in patients with chronic fatigue syndrome and depression: study protocol for a randomised controlled trial”	Facilitate new approaches in clinical practice and more meaningful results for patients experiencing states of chronic fatigue	Randomized clinical trial; level II
Anhedonia; Ito et al [31], 2019	“Augmentation of positive valence system-focused cognitive behavioral therapy by inaudible high-frequency sounds for anhedonia: a trial protocol for a pilot study”	Testing the effect of increasing inaudible high-frequency sounds on the effectiveness of CBT^a^ focused on the positive valence system to treat anhedonia	Pilot study; level II
Anhedonia; Braun et al [32], 2019	“A pilot study investigating the effect of music-based intervention on depression and anhedonia”	To investigate the effect of a music therapy-based approach to managing major depressive disorder and its associated symptoms.	Clinical trial; level III
Anhedonia; Ebrahem and Masry [33], 2017	“Effect of relaxation therapy on depression, anxiety, stress and quality of life among diabetic patients”	To evaluate the effect of relaxation therapy on major depressive disorder and its associated symptoms such as anxiety, stress, quality of life and blood glucose levels.	Quasi-experimental study; level II
Low self-esteem; Kinser et al [34], 2013	“‘A feeling of connectedness’: perspectives on a gentle yoga intervention for women with major depression”	To evaluate the feasibility, acceptability, and effects of yoga among women with major depressive disorder	Randomized clinical trial; level II
Low self-esteem; Grandia [35], 2014	“Patient-initiated strategies for self-management of depression and low mood: understanding theory and changing behavior”	Examine the theory of patient-planned behavior for major depressive disorder self-management	Randomized clinical trial; level II
Sleep disorders; Prathikanti et al [25], 2017	“Treating major depression with yoga: a prospective, randomized, controlled pilot trial”	Examine a therapeutic guideline of hatha yoga for 8 weeks as monotherapy for major depressive disorder	Randomized clinical trial; level II
Guilt; Sathyanarayan et al [36], 2019	“Role of yoga and mindfulness in severe mental illnesses: a narrative review”	To review the effectiveness of yoga and mindfulness as a treatment modality for major depressive disorder	Literature review; level V
Low self-esteem; van Grieken et al [37], 2015	“Patients’ perspective on self-management in the recovery from depression”	Determine therapeutic guidelines for patients to recover from major depressive disorder	Randomized clinical trial; level II
Sleep disorders; Ma et al [17], 2018	“The effects of Tai Chi on sleep quality in Chinese American patients with major depressive disorder: a pilot study”	Evaluate the effects of tai chi on sleep quality and functioning among patients with major depressive disorder	Pilot study; level II
Guilt; Sarsak et al [38], 2020	“Applied occupational therapy for major depressive disorder: clinical case report”	Describe an intervention carried out via occupational therapy on an older woman with major depressive disorder and suicidal thoughts	Clinical case study; level VI
Sleep disorders; Xu et al [18], 2023	“Clinical evidence for association of acupuncture with improved major depressive disorder: a systematic review and meta-analysis of randomized control trials”	To determine the efficacy and safety of acupuncture for major depressive disorder	Meta-analysis; level I
Angry mood; Souza [39], 2019	“Chinese auricular acupuncture in the treatment of depression”	To investigate the efficacy of Chinese auricular acupuncture for major depressive disorder	Study of a qualitative nature; level V
Sleep disorders; Farrar and Farrar [19], 2020	“Clinical aromatherapy”	Investigate the history, supporting theories, guidelines, plant sources, safety, and pathophysiological responses of clinical aromatherapy in nursing	Systematic literature review; level I
Guilt; Birgitta et al [40], 2018	“Treatment of depression and/or anxiety - outcomes of a randomised controlled trial of the tree theme method® versus regular occupational therapy”	To compare therapeutic guidelines for major depressive disorder in relation to activities of daily living, psychological symptoms of major depressive disorder, and health-related aspects	Randomized clinical trial; level II
Sleep disorders; Ter Heege et al [20], 2020	“The clinical relevance of early identification and treatment of sleep disorders in mental health care: protocol of a randomized control trial”	Identify the prevalence of sleep disorders in different mental disorders, including major depressive disorder	Meta-analysis; level I
Sleep disorders; Russi et al [27], 2022	“Physical therapy intervention in the treatment of insomnia”	To evaluate a tool for restoring physiological balance and improving sleep in major depressive disorder	Experimental study; level III
Guilt; Duggal [41], 2019	“Self-management of depression: beyond the medical model”	Describe a new self-management paradigm in major depressive disorder centered on the patient beyond the improvement of clinical symptoms	Systematic literature review; level I
Weight change; Naslund et al [42], 2017	“Lifestyle interventions for weight loss among overweight and obese adults with serious mental illness: a systematic review and meta-analysis”	Evaluate the effects of a therapeutic approach to lifestyle changes on short- and long-term changes in body weight in people with major depressive disorder	Systematic review and meta-analysis; level I
Angry mood; de Cassia Rondina et al [43], 2018	“Practicing physical exercise and symptoms of depression in college students”	To investigate the relationship between major depressive disorder symptoms and physical activity patterns in university students	Randomized clinical trial; level II
Weight change; Daumit et al [44], 2013	“A behavioral weight-loss intervention in persons with serious mental illness”	Determine the effectiveness of guidelines for weight loss in adults with severe mental illness, including major depressive disorder	Essay randomized clinical trial; level II
Appetite change; Kazemi et al [45], 2020	“Effect of probiotic and prebiotic vs placebo on psychological outcomes in patients with major depressive disorder: a randomized clinical trial”	Investigate the effect of supplementation with probiotics and prebiotics on appetite, BMI, weight, and energy intake in patients with major depressive disorder	Randomized clinical trial; level II
Weight change; Colombari et al [46], 2018	“The effect to behavioral weight-loss intervention on depressive symptoms among Latino immigrants in a randomized controlled trial”	Testing the effectiveness of therapeutic guidelines for changing lifestyle life versus usual care among adults with obesity and major depressive disorder	Systematic review and meta-analysis; level I
Weight change; Carraça et al [47], 2013	“The association between physical activity and eating self-regulation in overweight and obese women”	To assess the importance of physical exercise and healthy eating for managing low self-esteem and depressed mood in people with major depressive disorder	Randomized clinical trial; level II
Sleep disorders; Rethorst et al [21], 2015	“IL-1β and BDNF are associated with improvement in hypersomnia but not insomnia following exercise in major depressive disorder”	Examine changes in hypersomnia and insomnia after increased exercise in people with major depressive disorder	Randomized clinical trial; level II
Angry mood; de Vaz Pato Oom [48], 2019	“Physical activity in the treatment of young adult depressive disease”	To evaluate the beneficial effect of physical activity in the treatment of major depressive disorder	Literature review; level V
Low self-esteem; Kandola et al [49], 2019	“Physical activity and depression: towards understanding the antidepressant mechanisms of physical activity”	Evaluate the main biological and psychosocial mechanisms of activity as an antidepressant	Systematic review; level I
Low self-esteem; Barton et al [50], 2012	“Exercise-, nature- and socially interactive-based initiatives improve mood and self-esteem in the clinical population”	Evaluate initiatives to promote health through outdoor exercise involving a population with major depressive disorder	Randomized clinical trial; level II
Weight change; Soares et al [51], 2020	“Effects of physical exercise on obesity and depression: a review”	To integrate the findings on the effects of physical exercise on obesity and major depressive disorder	Literature review; level V
Anhedonia; Brush et al [52], 2022	“A randomized trial of aerobic exercise for major depression: examining neural indicators of reward and cognitive control as predictors and treatment targets”	To demonstrate the effectiveness of aerobic exercise among adults with major depressive disorder	Randomized controlled clinical trial; level II
Anhedonia; Belvederi et al [53], 2019	“Physical exercise in major depression: reducing the mortality gap while improving clinical outcomes”	Provide a concise update on the effectiveness of physical exercise on major depressive disorder and reduction in cardiovascular mortality	Systematic literature review; level I
Anhedonia; Turner et al [54], 2019	“Physical activity and depression in MS: the mediating role of behavioral activation”	Evaluate the impact of physical activity to improve fatigue and symptoms of depressive disorders in individuals with multiple sclerosis	Randomized controlled clinical trial; level II
Anhedonia; Toups et al [55], 2017	“Exercise is an effective treatment for positive valence symptoms in major depression”	To evaluate the effect of physical exercise on the symptoms of major depressive disorder	Essay randomized clinical trial; level II
Anhedonia; Archer et al [56], 2014	“Effects of physical exercise on depressive symptoms and biomarkers in depression”	To investigate the effects of physical exercise on depressive symptoms and biomarkers in major depressive disorder	Systematic review of the literature; level I
Angry mood; Bains and Abdijadid [57], 2022	“Major depressive disorder”	Identify, describe, and review the etiology, management, and clinical presentation of major depressive disorder	Review and meta-analysis; level I
Low self-esteem; Bajaj et al [58], 2016	“Mediating role of self-esteem on the relationship between mindfulness, anxiety, and depression”	To examine the mediating effects of self-esteem on the association among mindfulness, anxiety, and major depressive disorder	Cross-sectional study; level VI
Low self-esteem; Randal et al [59], 2015	“Mindfulness and self-esteem: a systematic review”	Evaluate studies investigating the association between mindfulness and self-esteem in relation to major depressive disorder	Systematic literature review; level I
Angry mood; Farb et al [60], 2018	“Prevention of relapse/recurrence in major depressive disorder with either mindfulness-based cognitive therapy or cognitive therapy”	Evaluate relapse rates in patients with depression in remission receiving CBT based on mindfulness and CT^b^	Meta-analysis; level I
Sleep disorders; Kuyken et al [22], 2019	“Efficacy of mindfulness-based cognitive therapy in prevention of depressive relapse: an individual patient data meta-analysis from randomized trials”	Conduct a meta-analysis of individual patient data to examine the effectiveness of mindfulness-based CT in the treatment of major depressive disorder	Systematic review and meta-analysis; level I
Appetite change; Katterman et al [61], 2014	“Mindfulness meditation as an intervention for binge eating, emotional eating, and weight loss: a systematic review”	Examine studies on mindfulness for the treatment of binge eating associated with major depressive disorder	Systematic review of the literature; level I
Guilt; Fawns [62], 2013	“Mindfulness based cognitive therapy and emotion focused therapy as treatments for major depression”	To describe the symptomatology and treatment options for major depressive disorder, and to examine and compare the effectiveness of two treatment orientations: mindfulness-based cognitive therapy and emotion-focused therapy	Literature review; level V
Angry mood; Economides et al [63], 2018	“Improvements in stress, affect and irritability following brief use of a mindfulness-based smartphone app: a randomized controlled trial”	To evaluate the mindfulness-based Headspace smartphone app for managing symptoms of major depressive disorder	Randomized clinical trial study; level II
Appetite change; Person [64], 2021	“A two-study investigation into the link between rumination and night eating, and symptom improvement following a mindfulness-based intervention”	To explore and investigate the link between night eating syndrome and depressive symptoms	Clinical study; level II
Sleep disorders; Bellingham [28], 2019	“Spiritually focused mindfulness meditation: an interpretative phenomenological analysis of the effect of spiritually focused mindfulness meditation on depression with a clinical population”	Explore the use of focused mindfulness meditation for major depressive disorder	Qualitative study; level VI
Guilt; Schanche et al [65], 2020	“The effects of mindfulness-based cognitive therapy on risk and protective factors of depressive relapse - a randomized wait-list controlled trial”	Explore the effects of mindfulness-based CT on risk and protective factors for major depressive disorder relapse in the domains of cognition, emotion, and self-relationship	Randomized clinical trial study; level II
Angry mood; Rech et al [66], 2022	“Techniques for managing the emotion of anger: a systematic review”	Identify the main techniques for managing anger in adults with major depressive disorder	Systematic review of the literature; level I
Sleep disorders; Chung et al [23], 2020	“Mobile app use for insomnia self-management in urban community-dwelling older Korean adults: retrospective intervention study”	Explore the relationship between sleep quality, memory concerns (memory loss) and depressive symptoms	Randomized clinical trial; level II
Low self-esteem; Moloud et al [67], 2022	“Cognitive-behavioral group therapy in major depressive disorder with focus on self-esteem and optimism: an interventional study”	Determine the effect of group CBT for the management of low self-esteem in patients with major depressive disorder	Randomized clinical trial study; level II
Guilt; Dobkin et al [68], 2019	“Cognitive behavioral therapy improves diverse profiles of depressive symptoms in Parkinson’s disease”	Examine the impact of CBT on different depressive symptoms in Parkinson disease	Randomized clinical trial; level II
Angry mood; Santos [69], 2017	“Efficacy of procedural cognitive therapy and behavioral activation in the treatment of major depressive disorder: a randomized clinical trial”	To compare the effectiveness of psychotherapies for patients with major depressive disorder	Randomized clinical trial study; level II
Angry mood; Ahern et al [70], 2017	“Clinical efficacy and economic evaluation of online cognitive behavioral therapy for major depressive disorder: a systematic review and meta-analysis”	Evaluate the clinical effectiveness and evidence for the use of online CBT as an affordable treatment solution for major depressive disorder	Systematic review and meta-analysis; level I
Low self-esteem; Korrelboom et al [71], 2012	“Competitive memory training (COMET) for treating low self-esteem in patients with depressive disorders: a randomized clinical trial”	Evaluate whether competitive memory training is an effective intervention for patients with major depressive disorder	Randomized clinical trial study; level II
Anhedonia; Wang et al [72], 2020	“Guided self-help behavioral activation intervention for geriatric depression: protocol for pilot randomized controlled trial”	Pilot a therapeutic guided self-help intervention for the treatment of major depressive disorder in older adults	Randomized clinical trial; level II
Guilt; Alves and Bonvicini [73], 2022	“The role of behavioral activation in the management of depressive symptoms”	Describe and evaluate behavioral activation as a psychotherapy tool and guideline for managing mood, behavior, and emotions in patients with major depressive disorder	Systematic review; level I
Fatigue; Wisenthal et al [74], 2019	“Insights into cognitive work hardening for return-to-work following depression: qualitative findings from an intervention study”	Contribute to the literature on the effectiveness of cognitive maturation to return to work after episodes of major depressive disorder	Randomized clinical trial; level II
Weight changes; Berman et al [75], 2016	“Uncontrolled pilot study of an acceptance and commitment therapy and health at every size intervention for obese, depressed women: accept yourself!”	Evaluate the feasibility and outcomes of a new treatment based on self-acceptance for women with obesity and major depressive disorder	Randomized uncontrolled clinical trial; level II

^a^CBT: cognitive behavioral therapy.

^b^CT: cognitive therapy.

For the symptom of anhedonia, 9 publications were selected: 3 (33%) published on BVS [32,52,72], 3 (33%) published on PubMed [53-55], 2 (22%) published on Scopus [31,56], and 1 (11%) published as gray literature [33]. Similarly, the symptom of low self-esteem was identified in 9 publications: 5 (56%) published in PubMed [34,37,49,50,71], 1 (11%) published on Scopus [58], and 3 (33%) published as gray literature [35,59,67]. Furthermore, 9 publications were also selected for the symptom of irritated mood: 4 (44%) published on PubMed [57,60,63,70] and 5 (56%) published as gray literature [39,43,48,66,69].

For the symptom of guilt, 8 publications were selected: 4 (50%) published on PubMed [40,41,65,68] and 4 (50%) published as gray literature [36,38,62,73]. Regarding the symptom of weight change, 6 publications were identified: 5 (83%) published on PubMed [42,44,46,47,75] and 1 (17%) published as gray literature [51]. For the symptom of fatigue, 3 publications were selected: 2 (67%) published on PubMed [29,30] and 1 (33%) published on CINAHL [74]. Finally, for the symptom of appetite change, 3 publications were identified: 2 (67%) published on PubMed [45,61] and 1 (33%) published as gray literature [64]. Regarding the level of evidence, 48% (30/62) of the studies were level II, 34% (21/62) were level I, 8% (5/62) were level V, 6% (4/62) were level VI, and 3% (2/62) were level III.

### Self-Management of Major Depressive Disorder

The evidence available from the included studies to promote self-management of the symptoms of major depressive disorder describes the characteristics, benefits, and applicability of therapeutic guidelines in everyday life, demonstrating that these guidelines can promote a safe space to share conversations on sensitive subjects such as stigma and prejudice, adverse drug events, diagnosis, and relapse [14-75].

The findings also point out that, to promote self-management through therapeutic guidelines, it is necessary to know the functionality of the patient or family, have access to community resources and a social support network, and monitor the participation of these individuals in their care [16,33,35,37,40]. The interdisciplinary team’s online or face-to-face follow-up of patients with major depressive disorder involves monitoring the use of therapeutic guidelines and whether they are being carried out correctly and appropriately considering the individual’s uniqueness, as well as monitoring changes in symptoms and problems with medication, providing social support, and identifying patients at high risk of relapse [23,41].

Another common point cited in the studies was the ability of health professionals to instruct patients with major depressive disorder on how to deal with symptoms and biosociopsychological demands [16,31-33]. Professionals require skills and good communication with patients when instructing them on the use of therapeutic guidelines for managing the disorder, since incorrect instructions or misunderstandings may make the application of therapeutic guidelines less efficient [23,35,37,40,67,70,74,75]. If the professional advises the patient to sunbathe (phototherapy) during the day, but does not specify the time, this may cause the patient to take this guidance at inappropriate times and be harmful to their health. Therefore, phototherapy would not be effective. The findings encourage a multidisciplinary approach to the applicability of therapeutic guidelines, focusing on the specificities of each person and collective actions, strengthening the patient’s ability to self-manage.

### Therapeutic Guidelines for Self-Management

The analysis of the studies, as described previously, resulted in the mapping and categorization of 7 large groups of therapeutic guidelines for the self-management of major depressive disorder. These guidelines are made up of a set of actions or activities (n=40), which have been called subinterventions based on the similarities between them (Table 3).

In total, 52% (32/62) of the therapeutic guidelines identified fell into the category of psychotherapy, which helps monitor and plan activities with the person (in the context of their symptoms), manage adverse experiences, and develop social skills [14,16,17,22,23,26,28,29,31,36-38,46,56,58-72,74,75]. Adoption of healthy habits (25/62, 40%) and integrative and complementary practices (17/62, 27%) were the second and third most observed categories. The process of adopting integrative and complementary practices [17,19,20,24-26,29-40], together with healthy habits such as eating a balanced diet and exercising frequently and for the right duration, showed a good response in improving depressive conditions, as well as being an important factor in preventing relapses of major depressive disorder [21,26,27,29,30,33,37,38,41-57].

Finally, relaxation techniques (9/62, 14%), consultation with a health professional (14/62, 22%), pharmacological therapy (9/62, 14%), and leisure or pleasurable activities (4/62, 6%) were the least observed therapeutic guidelines in the studies. However, they offered promising insights into self-management of the disorder and underscored the importance of a multifaceted approach to managing this complex condition [16,20,23, 26-30,33,36-38,41,50,57,66].

It is widely recognized that the concept of self-management is traditionally associated with approaches that the patient can carry out autonomously, such as adopting healthy habits, physical exercise, and relaxation techniques. However, it is crucial to emphasize that, in the context of major depressive disorder, self-management transcends these independent symptom management practices [37].

It involves empowering the patient to take active control and gain an in-depth understanding of their condition through the application of a variety of therapeutic interventions [35]. Patients with major depressive disorder may experience feelings of frustration and a range of negative thoughts, see everyday problems as major catastrophes, have difficulty recognizing efforts that awaken hope for life, and cope with the possible adverse events that psychiatric medications can cause [66].

For this reason, orientation toward psychotherapy and psychopharmaceuticals is justified as a self-management strategy as a way of coping with the adverse effects of medication and all the feelings of self-demand, excessive demands, and frustration caused by the disorder.

In relation to the population to which this study refers, the results show that patients with major depressive disorder have motivational, cognitive, and psychological deficits that can modify their ability to cope and reason [22,60,63,65].

In view of this, self-management of the signs and symptoms resulting from major depressive disorder needs to take place at the beginning of treatment, with the support of family or caregivers; friends; community organizations; and, most especially, the multi-professional health team to help with the correct use of therapeutic options until the person is able to self-manage independently [14-75]. Therefore, the therapeutic guidelines found in this review are not restricted to the professional-patient relationship; these relationships are broad and include families and the community.

**Table 3:** Mapping of the results regarding the therapeutic guidelines.

Therapeutic guideline	Subinterventions
Adopting healthy habits	Physical activity or regular exercise [21,26,27,29,30,33,37,38,41-57] Healthy eating [26,27,29,37,41-46]
Leisure or pleasurable activities	Listening to music, watching movies, reading, painting, going out with friends, taking outdoor walks, and practicing spiritual and religious activities [37,38,41,50]
Relaxation techniques	Progressive muscle relaxation [33,41,66] Respiratory rehabilitation [16,26,27,29,30,36] Stretching [16,26,27,29,41,66] Warm baths [16] Massages [29,41,66] Meditation [30]
Pharmacological therapy	Psychopharmacological therapy [16,20,26,28,29,36,38,41,57]
Consultation with a health professional	Psychoeducation or professional counseling [16,20,23,26,27,29,36-38,41-43,57,66]
Psychotherapy	Behavioral activation [69,73] Cognitive behavioral psychotherapy [14,16,23,26,29,31,38,58,67-71] Mindfulness [22,26,28,36-38,59-66] Psychotherapy focused on problem-solving [29,38,74] Competitive memory training psychotherapy [71] Self-efficacy psychotherapy [46] Guided self-help psychotherapy [23,72] Interpersonal psychotherapy [17,23,56] Therapy focused on acceptance, commitment [62], and emotion [75]
Integrative and complementary practices	Acupuncture [19,26,29,39] Music therapy [24,30-33] Phytotherapy [26,29] Phototherapy [26,29] Aromatherapy [19] Cryotherapy [29] Thermotherapy [29] Tai chi [17,29,38] Reflexology [29] Qigong [29] Yoga [25,29,34-37] Art therapy [40] Chromotherapy [20]

## Discussion

### Principal Findings

From this review, it was possible to identify the therapeutic guidelines for the self-management of the symptoms of major depressive disorder in scientific production as psychotherapy, adoption of healthy habits, integrative and complementary practices, relaxation techniques, consultation with a health professional, pharmacological therapy, and leisure or pleasurable activities.

The selected studies show that knowledge on the subject significantly improves self-management of the symptoms of major depressive disorder through these therapeutic guidelines to promote behavior changes and awareness of the symptoms of the disorder, prevention of relapses, reduction of the perception of obstacles, and increased adherence to treatment.

A small number of articles published in journals on the subject were found for all the symptoms investigated, especially irritable mood, followed by fatigue, weight change, appetite change, sleep changes, and low self-esteem, necessitating the addition of secondary documents that considerably broadened the identification of other therapeutic guidelines.

Most of the studies originated in America (40/62, 63%), where the largest number of people with major depressive disorder is concentrated. Regarding the concept that triggered the research, the studies involved health professionals, students, and the general population, covering the definition, description, comparative analysis, efficiency, and applicability of therapeutic guidelines. These studies were included to increase the possibility of nurses playing a leading role in the self-management of symptoms of major depressive disorder [14-75].

The factors considered important for the self-application of therapeutic guidelines in the studies were cognition; level of education; environmental factors; and the functional capacity of the patient, caregivers, and family members. It should be emphasized that, for self-management, clients who are going to follow these therapeutic guidelines should receive them in clear and objective language and format, with specificity and relevance for each symptom, enabling adherence to treatment and favoring the therapist–patient or caregiver bond [26]. These therapeutic guidelines are also considered low cost and easy to use, optimize rehabilitation and autonomy, and encourage self-care [26]. It is important to highlight the definitions of each of the therapeutic guidelines identified in this review.

Integrative and complementary practices, as therapeutic resources based on traditional knowledge, were the therapeutic guidelines most commonly found in the literature as effective for the self-management and management of major depressive disorder symptoms, and their applicability aimed to induce a state of harmony and balance throughout the body.

Phototherapy has been shown to reduce depressive symptoms and improve irregularities in sleep patterns and quality, especially in older adults with major depressive disorder, as long as it is used with a certain frequency and is administered before 10 AM [14-16,26]. It has also been used in the control of nocturnal hyperphagia and has shown reductions in the percentage of food eaten after dinner and in the number of nocturnal meals eaten per week, reducing the rate of overweight and obesity, as well as being used in the control of fatigue, where it has shown a reduction in the feeling of tiredness [29].

Music therapy applied to adults [24] has been shown to be effective in physical and mental relaxation, reducing anxious and depressive symptoms and promoting well-being in a conscious and healthy way [30]. Listening to soothing noises and classical music associated with rhythmic breathing produced a significantly higher rate of adherence to major depressive disorder treatment, especially in the patients with the highest severity [31-33].

A study has shown that anhedonia and clinically significant depressive symptoms can be resistant to standard treatment but adjuvant treatment with high-frequency inaudible sound therapy increases the reward of related brain circuits and has a synergistic effect on anhedonia [31]. Integrating music therapy with conventional major depressive disorder treatment (therapies and medication) gives people the opportunity to get in touch with their emotions and provides distraction and a means of communication capable of overcoming barriers and limits to verbal expression [30].

Yoga [29,34,35] has been a beneficial exercise for managing sleep quality and other symptoms of major depressive disorder [25,36]. It is an important tool for promoting self-care and, consequently, care for everything around the patient [37]. A study carried out to explore the experiences of patients with major depressive disorder showed that practicing yoga as a self-management strategy helped them gain better insights into their own condition, improving the quality of their health care [37].

Tai chi has been shown to be more effective than traditional rehabilitation (psychotropic drugs and therapies) in relation to insomnia, with an improvement in the quality, duration, and efficiency of sleep and a reduction in thoughts of guilt associated with major depressive disorder [17,29,38]. Acupuncture has been shown to be effective in treating people with major depressive disorder [18,39], mainly in reducing sleep disorders but also in symptoms related to changes in appetite, irritability, anxiety, and sadness, and has not been shown to be effective for suicidal ideation [26,29].

With regard to herbal medicines, St John’s wort, *chai hu*, and *gancao* were the most widely used. In 3% (2/62) of the studies, after 2 weeks of administering these herbs (alone or in combination) as an alternative to conventional antidepressants, they produced a statistically significant improvement in symptoms of altered sleep [26,29]. Although the effects of these herbs are not fully understood, it is likely that they produce antidepressant effects through multiple pathways or targets that interact with each other.

To a lesser extent among the studies but no less importantly, aromatherapy [19], cryotherapy, thermotherapy, reflexology, qigong [29], art therapy [40], and chromotherapy [20] improved empowerment in the search for self-care and taking responsibility for one’s own health, as well as reducing levels of anxiety and stress, improving sleep disorders, improving the immune system, and lowering blood pressure levels [29].

People with major depressive disorder have shown improvements in sleep disorders, anxiety, and stress, as well as enhancements in immune system function and reductions in blood pressure levels when using respiratory rehabilitation [16,26,27,29,30,36], stretching [16,26,27,29,41,66], warm baths [16], massages [29,41,66], acupuncture, herbal medicine, phototherapy, reflexology, qigong, and yoga [17,19,20,24,26,29,34,38-40] as therapeutic guideline.

Cryotherapy and thermotherapy are not commonly used to treat the symptoms of major depressive disorder, but they help relax the body and mind as a physiotherapeutic resource [16,29]. There was a significant improvement in stress and body pain related to fatigue, anxiety, and swelling in the feet and legs [29].

Another important therapeutic guideline was the adoption of healthy lifestyle habits, such as healthy eating [17,26,27,29,41-46] and physical exercise [21,26,27,30,33,37,38,41-57]. These reduce stress; improve mood, body image perception, and self-esteem; stimulate cognitive functioning; promote greater satisfaction with life [45,46]; and can help people feel stronger and more capable, helping reduce the feelings of hopelessness and helplessness associated with major depressive disorder [53,54].

It should be noted that, regardless of the physical activity chosen, people with major depressive disorder need to make it part of their routine and do it [47] according to their tolerance and state of health [54]. An important concern in relation to physical activity for people with major depressive disorder is the fact that some of the common symptoms of depression (fatigue, lack of energy, psychomotor retardation, despair, and feelings of worthlessness) interfere with the motivation to exercise [57].

In more severe cases, practicing physical activity can be difficult for people with major depressive disorder [55]. This can compromise adherence and long-term permanence in the exercise program [52]. It is recommended to prescribe structured exercises based on the activities already practiced by the patient to reduce obstacles [33].

Another group of therapeutic guidelines that were also commonly identified in this review were psychotherapies. Mindfulness-based therapy [58,59] is a promising therapeutic approach for treating major depressive disorder and is part of the list of integrative and complementary practices [26,37,60]. It combines elements of therapeutic guidelines [22,61] with the aim of helping people develop a full awareness of the present moment, accepting and acknowledging their thoughts, emotions, and bodily sensations without judgment [62] and learning to observe their negative thoughts and dysfunctional thought patterns without allowing themselves to be involved with or controlled by them [63].

Mindfulness improves self-management of prodromal symptoms [64], represents a stability factor when practiced frequently and in a supervised manner [28], positively influences memory and distorted feelings of guilt, increases cognitive resilience, balances mood and sleep, reduces high levels of stress, and improves self-esteem and anxiety [65] associated with major depressive disorder relapse.

Similarly, cognitive behavioral psychotherapy [26,66] uses positive reinforcement techniques and systematic desensitization, helping people cope with stress, face the challenges of everyday life, change negative thought patterns and behaviors to more realistic and positive ones [23,67], and identify the first signs of relapse and prevent it as it focuses on social skill training [68,69]. Recently, online cognitive behavioral psychotherapy has been shown to be effective for depressed and angry mood as a cost-effective treatment modality for major depressive disorder [70].

Other psychotherapies aimed at influencing the person with major depressive disorder and helping them modify emotional, cognitive, and behavioral problems were self-efficacy psychotherapy [46]; competitive memory training [71]; guided self-help psychotherapy [23,72]; behavioral activation [69,73]; interpersonal psychotherapy [38,57,67]; psychotherapy focused on problem-solving [29,38,74]; and therapy focused on acceptance, commitment, and emotion [62,75].

Leisure time or pleasurable activities as a social practice, contrary to what is often thought, are not only carried out during the summer or on vacation but also in between daily obligations [37]. Thus, they can take the form of individual or group dynamics, whether it is reading a book, listening to music, or going for a walk with friends [37,50]. The relationship between leisure and health has fostered new techniques for the treatment of depressive disorders with the aim of enabling the individual’s psychic and social readaptation [37,50].

Painting for leisure, for example, can promote the manifestation of feelings through emotions expressed verbally or not, thus helping understand the mind and its sorrows [38]. In this sense, of the possibilities for leisure activities recommended by the professionals who collaborated with the data found, art therapy [40], group and individual outings [41,50], trips [38], reading books and watching movies [37,38], listening to music [31], and attending religious institutions [50] were the most cited in the studies.

In the context of pharmacological treatment, antidepressants associated with psychotherapies [26,29,37] should be administered with caution and under medical supervision [20,38,41] based on the therapeutic alliance, clinical history, monitoring and reassessment of psychiatric conditions, and adequacy of the diagnosis, and guidance should be provided for families [28,57].

In 5% (3/62) of the studies, the association of psychotropic drugs with therapeutic guidelines was highlighted (due to the severity of the major depressive disorder clinical picture) even in the face of difficulties with medication adherence for different reasons [16,29,38], which should be anticipated and addressed proactively in every contact with the patient. Approximately 30% to 40% of people using antidepressants still do not respond as expected to treatment [27,41].

Relaxation techniques appeared in this review as a more appropriate way of coping with the psychological, environmental, and social stimuli that people with major depressive disorder may face. Among these strategies, breathing techniques [16,26,27,29,30,36], progressive muscle relaxation [33,41,66], massages [29,41,66], stretching [16,26,27,29,66], a warm bath [16], and meditation [30] were the most cited.

In the context of major depressive disorder, these techniques promote muscle relaxation, relieve stress and tension, rebalance emotions, produce hormones such as endorphins, provide a sense of well-being, and significantly improve sleep quality [16]. Incorporating these practices throughout the day or even before going to bed helps people let go of tiredness, become aware of the present moment, and pay attention to their own bodies [16,66].

Finally, therapeutic guidelines for psychoeducation or professional counseling can be used to help with self-management as major depressive disorder generates changes in the family system and structure [16,18,26,36,38], resulting in the need for clarification to relieve anxieties and doubts to improve the psychological well-being of the patient [20,37,42,43].

Thus, the wide variety of therapeutic guidelines found can increase knowledge about the symptoms and aspects of major depressive disorder, allowing the person to self-manage symptoms such as fatigue, sleep disturbances, anhedonia, and mood swings [16,27,29,66] in a more responsible and autonomous way.

These therapeutic guidelines are characterized by providing information on the diagnosis, etiology, prognosis, and course of the illness; the identification of early signs of crisis; the importance of adherence to medication or psychosocial treatment; the promotion of healthy habits and regularity in lifestyle (sleep, diet, physical activity, and substance use); and how to deal with the stigmatization, doubts, fears, and myths regarding major depressive disorder [23,41,42].

Therefore, the implications for practice are based on the fact that therapeutic guidelines help in the self-management of major depressive disorder through behavioral, social, and emotional changes that allow for better construction of clinical reasoning, adaptation, autonomy, coping, and improvement of general health.

### Limitations

The limitations of this scoping review are related to the nature of the review itself as its aim was to provide an overview of therapeutic guidelines, which may not be sufficient for the self-management of major depressive disorder as it is a complex clinical condition with a heterogeneity of symptoms. Furthermore, despite efforts to develop a comprehensive search strategy, it was difficult to find controlled and uncontrolled descriptors for the term “therapeutic guidelines,” which is not an indexed descriptor.

### Conclusions

By analyzing the methodological approach adopted in this study, and in accordance with the proposed objectives, it was possible to highlight a set of therapeutic guidelines to support a person’s self-management in the context of major depressive disorder symptoms. This does not preclude further study of their effectiveness in the self-management of major depressive disorder symptoms but demonstrates their contribution to promoting self-management of major depressive disorder and their power to prevent possible complications.

Self-management is a promising strategy that emphasizes the person’s responsibility in the care process. It goes beyond participation in health care interactions and includes dealing with symptoms and disability; managing medication and monitoring indicators; maintaining adequate levels of nutrition and exercise; and adjusting to psychological, social, and lifestyle demands.

These therapeutic guidelines can be applied by all members of a multi-professional mental health team, including nurses, provided they are trained as resources to help the person acquire new knowledge, behaviors, and skills for self-management. These guidelines are also an important resource for health professionals in their role as facilitators of a person’s development of knowledge and skills to ensure their capacity for self-management.

## Appendix

Multimedia Appendix 1 PRISMA (Preferred Reporting Items for Systematic Reviews and Meta-Analyses extension for Scoping Reviews) checklist.

Multimedia Appendix 2 Search strategy.

## Abbreviations

JBI: Joanna Briggs Institute
PCC: population, concept, and context
PRISMA: Preferred Reporting Items for Systematic reviews and Meta-Analyses
PRISMA-ScR: Preferred Reporting Items for Systematic Reviews and Meta-Analyses extension for Scoping Reviews

## Data Availability

All data generated or analyzed during this study are included in this published article (and its supplementary information files).
