# Supplementary material for: Therapeutic Guidelines for the Self-Management of Major Depressive Disorder: Scoping Review
Source: Interact J Med Res. 2025 Mar 6;14:e63959. doi: 10.2196/63959 (PMC11926446; doi:10.2196/63959)
Supplement: Multimedia Appendix 2 [file ijmr_v14i1e63959_app2.docx]

**Multimedia Appendix 2**

A single strategy was adapted for all data sources, maintaining similarities in descriptor combinations as shown below.

Search strategy for retrieving studies.

| #1 | Strategy adopted |
| --- | --- |
| Lilacs® | ("depressive disorder, major" OR "major depression"[All Fields]) AND ("Self Care") OR ("Self efficacy") OR ("Self-efficacy") OR ("Self-care") OR ("Self control") OR ("Self-care Behavior") OR ("Self care Behavior") OR ("Self-care Strategies") OR ("Self care Strategies") OR (MH "Self concept") OR ("Self-concept") OR ("Self-regulation") OR ("Self regulation") OR ("Self management") OR ("Self-management") OR ("Self care agency") OR ("Self-care agency") OR ("Self care demand") OR ("Self-care demand") OR ("Self care requisites") OR ("Self-care requisites") OR ("Self monitotoring") OR ("Self-monitotoring") OR ("Self-Medication") OR ("Symptom management") OR ("Patient autonomy*") OR ("Patient Compliance") OR ("Health behavior") OR ("Attitude to health") OR ("Attitude to illness") OR ("Patient attitude*") OR ("Choice behavior") OR ("Illness behavior") AND ("weight loss" OR "weight gain" OR "appetite" AND ("anhedonia") AND ("irritable mood") AND ("self-esteem") AND ("guilt"). |
| PubMed® | ("depressive disorder, major"[MeSH Terms] OR "major depression"[All Fields]) AND (MH "Self-care") OR(MH "Self-efficacy") OR ("Self-control") OR ("Self-care Behavior") OR ("Self-care Strategies") OR (MH "Self-concept") OR ("Self-regulation") OR ("Self-management") OR ("Self-careagency") OR ("Self-care demand") OR ("Self-care requisites") OR ("Self-monitoring") OR OR ("Self- medication") OR ("Symptom management") OR ("Patient autonomy*") OR (MH "Patient Compliance") OR (MH "Health behavior") OR (MH "Attitude to health") OR ("Attitude to illness") OR ("Patient attitude*") OR (MH "Choice behavior") OR (MH "Illness behavior") AND (intervention) AND ("insomnia" OR "hypersomnia" AND ("fatigue") AND ("weight loss" OR "weight gain" OR "appetite" AND ("anhedonia") AND ("irritable mood") AND ("self-esteem") AND ("guilt"). |
| Scielo® | ("depressive disorder, major"[MeSH Terms] OR "major depression"[All Fields]) AND (MH "Self-care") OR(MH "Self-efficacy") OR ("Self-control") OR ("Self-care Behavior") OR ("Self-care Strategies") OR (MH "Self-concept") OR ("Self-regulation") OR ("Self-management") OR ("Self-careagency") OR ("Self-care demand") OR ("Self-care requisites") OR ("Self-monitoring") OR OR ("Self- medication") OR ("Symptom management") OR ("Patient autonomy*") OR (MH "Patient Compliance") OR (MH "Health behavior") OR (MH "Attitude to health") OR ("Attitude to illness") OR ("Patient attitude*") OR (MH "Choice behavior") OR (MH "Illness behavior") AND (intervention) AND ("insomnia" OR "hypersomnia" AND ("fatigue") AND ("weight loss" OR "weight gain" OR "appetite" AND ("anhedonia") AND ("irritable mood") AND ("self-esteem") AND ("guilt"). |
| Scopus® | ("depressive disorder, major"[MeSH Terms] OR "major depression"[All Fields]) AND (MH "Self-care") OR(MH "Self-efficacy") OR ("Self-control") OR ("Self-care Behavior") OR ("Self-care Strategies") OR (MH "Self-concept") OR ("Self-regulation") OR ("Self-management") OR ("Self-careagency") OR ("Self-care demand") OR ("Self-care requisites") OR ("Self-monitoring") OR OR ("Self- medication") OR ("Symptom management") OR ("Patient autonomy*") OR (MH "Patient Compliance") OR (MH "Health behavior") OR (MH "Attitude to health") OR ("Attitude to illness") OR ("Patient attitude*") OR (MH "Choice behavior") OR (MH "Illness behavior") AND (intervention) AND ("insomnia" OR "hypersomnia" AND ("fatigue") AND ("weight loss" OR "weight gain" OR "appetite" AND ("anhedonia") AND ("irritable mood") AND ("self-esteem") AND ("guilt"). |
| Web of Science® | ("depressive disorder, major"[MeSH Terms] OR "major depression"[All Fields]) AND (MH "Self-care") OR(MH "Self-efficacy") OR ("Self-control") OR ("Self-care Behavior") OR ("Self-care Strategies") OR (MH "Self-concept") OR ("Self-regulation") OR ("Self-management") OR ("Self-careagency") OR ("Self-care demand") OR ("Self-care requisites") OR ("Self-monitoring") OR OR ("Self- medication") OR ("Symptom management") OR ("Patient autonomy*") OR (MH "Patient Compliance") OR (MH "Health behavior") OR (MH "Attitude to health") OR ("Attitude to illness") OR ("Patient attitude*") OR (MH "Choice behavior") OR (MH "Illness behavior") AND (intervention) AND ("insomnia" OR "hypersomnia" AND ("fatigue") AND ("weight loss" OR "weight gain" OR "appetite" AND ("anhedonia") AND ("irritable mood") AND ("self-esteem") AND ("guilt"). |
| BDENF® | ("depressive disorder, major"[MeSH Terms] OR "major depression"[All Fields]) AND (MH "Self-care") OR(MH "Self-efficacy") OR ("Self-control") OR ("Self-care Behavior") OR ("Self-care Strategies") OR (MH "Self-concept") OR ("Self-regulation") OR ("Self-management") OR ("Self-careagency") OR ("Self-care demand") OR ("Self-care requisites") OR ("Self-monitoring") OR OR ("Self- medication") OR ("Symptom management") OR ("Patient autonomy*") OR (MH "Patient Compliance") OR (MH "Health behavior") OR (MH "Attitude to health") OR ("Attitude to illness") OR ("Patient attitude*") OR (MH "Choice behavior") OR (MH "Illness behavior") AND (intervention) AND ("insomnia" OR "hypersomnia" AND ("fatigue") AND ("weight loss" OR "weight gain" OR "appetite" AND ("anhedonia") AND ("irritable mood") AND ("self-esteem") AND ("guilt"). |
| Cinahl® | ("depressive disorder, major"[MeSH Terms] OR "major depression"[All Fields]) AND (MH "Self-care") OR(MH "Self-efficacy") OR ("Self-control") OR ("Self-care Behavior") OR ("Self-care Strategies") OR (MH "Self-concept") OR ("Self-regulation") OR ("Self-management") OR ("Self-careagency") OR ("Self-care demand") OR ("Self-care requisites") OR ("Self-monitoring") OR OR ("Self- medication") OR ("Symptom management") OR ("Patient autonomy*") OR (MH "Patient Compliance") OR (MH "Health behavior") OR (MH "Attitude to health") OR ("Attitude to illness") OR ("Patient attitude*") OR (MH "Choice behavior") OR (MH "Illness behavior") AND (intervention) AND ("insomnia" OR "hypersomnia" AND ("fatigue") AND ("weight loss" OR "weight gain" OR "appetite" AND ("anhedonia") AND ("irritable mood") AND ("self-esteem") AND ("guilt"). |
| Ageline® | ("depressive disorder, major"[MeSH Terms] OR "major depression"[All Fields]) AND (MH "Self-care") OR(MH "Self-efficacy") OR ("Self-control") OR ("Self-care Behavior") OR ("Self-care Strategies") OR (MH "Self-concept") OR ("Self-regulation") OR ("Self-management") OR ("Self-careagency") OR ("Self-care demand") OR ("Self-care requisites") OR ("Self-monitoring") OR OR ("Self- medication") OR ("Symptom management") OR ("Patient autonomy*") OR (MH "Patient Compliance") OR (MH "Health behavior") OR (MH "Attitude to health") OR ("Attitude to illness") OR ("Patient attitude*") OR (MH "Choice behavior") OR (MH "Illness behavior") AND (intervention) AND ("insomnia" OR "hypersomnia" AND ("fatigue") AND ("weight loss" OR "weight gain" OR "appetite" AND ("anhedonia") AND ("irritable mood") AND ("self-esteem") AND ("guilt"). |
| Cochrane® | ("depressive disorder, major"[MeSH Terms] OR "major depression"[All Fields]) AND (MH "Self-care") OR(MH "Self-efficacy") OR ("Self-control") OR ("Self-care Behavior") OR ("Self-care Strategies") OR (MH "Self-concept") OR ("Self-regulation") OR ("Self-management") OR ("Self-careagency") OR ("Self-care demand") OR ("Self-care requisites") OR ("Self-monitoring") OR OR ("Self- medication") OR ("Symptom management") OR ("Patient autonomy*") OR (MH "Patient Compliance") OR (MH "Health behavior") OR (MH "Attitude to health") OR ("Attitude to illness") OR ("Patient attitude*") OR (MH "Choice behavior") OR (MH "Illness behavior") AND (intervention) AND ("insomnia" OR "hypersomnia" AND ("fatigue") AND ("weight loss" OR "weight gain" OR "appetite" AND ("anhedonia") AND ("irritable mood") AND ("self-esteem") AND ("guilt"). |
| B-on® | ("depressive disorder, major"[MeSH Terms] OR "major depression"[All Fields]) AND (MH "Self-care") OR(MH "Self-efficacy") OR ("Self-control") OR ("Self-care Behavior") OR ("Self-care Strategies") OR (MH "Self-concept") OR ("Self-regulation") OR ("Self-management") OR ("Self-careagency") OR ("Self-care demand") OR ("Self-care requisites") OR ("Self-monitoring") OR OR ("Self- medication") OR ("Symptom management") OR ("Patient autonomy*") OR (MH "Patient Compliance") OR (MH "Health behavior") OR (MH "Attitude to health") OR ("Attitude to illness") OR ("Patient attitude*") OR (MH "Choice behavior") OR (MH "Illness behavior") AND (intervention) AND ("insomnia" OR "hypersomnia" AND ("fatigue") AND ("weight loss" OR "weight gain" OR "appetite" AND ("anhedonia") AND ("irritable mood") AND ("self-esteem") AND ("guilt"). |
| BVS® | ("depressive disorder, major"[MeSH Terms] OR "major depression"[All Fields]) AND (MH "Self-care") OR(MH "Self-efficacy") OR ("Self-control") OR ("Self-care Behavior") OR ("Self-care Strategies") OR (MH "Self-concept") OR ("Self-regulation") OR ("Self-management") OR ("Self-careagency") OR ("Self-care demand") OR ("Self-care requisites") OR ("Self-monitoring") OR OR ("Self- medication") OR ("Symptom management") OR ("Patient autonomy*") OR (MH "Patient Compliance") OR (MH "Health behavior") OR (MH "Attitude to health") OR ("Attitude to illness") OR ("Patient attitude*") OR (MH "Choice behavior") OR (MH "Illness behavior") AND (intervention) AND ("insomnia" OR "hypersomnia" AND ("fatigue") AND ("weight loss" OR "weight gain" OR "appetite" AND ("anhedonia") AND ("irritable mood") AND ("self-esteem") AND ("guilt"). |
| Ibecs® | ("depressive disorder, major"[MeSH Terms] OR "major depression"[All Fields]) AND (MH "Self-care") OR(MH "Self-efficacy") OR ("Self-control") OR ("Self-care Behavior") OR ("Self-care Strategies") OR (MH "Self-concept") OR ("Self-regulation") OR ("Self-management") OR ("Self-careagency") OR ("Self-care demand") OR ("Self-care requisites") OR ("Self-monitoring") OR OR ("Self- medication") OR ("Symptom management") OR ("Patient autonomy*") OR (MH "Patient Compliance") OR (MH "Health behavior") OR (MH "Attitude to health") OR ("Attitude to illness") OR ("Patient attitude*") OR (MH "Choice behavior") OR (MH "Illness behavior") AND (intervention) AND ("insomnia" OR "hypersomnia" AND ("fatigue") AND ("weight loss" OR "weight gain" OR "appetite" AND ("anhedonia") AND ("irritable mood") AND ("self-esteem") AND ("guilt"). |
|  | Gray Literature |
| Google Scholar® | ("depressive disorder, major"[MeSH Terms] OR "major depression"[All Fields]) AND (MH "Self-care") OR(MH "Self-efficacy") OR ("Self-control") OR ("Self-care Behavior") OR ("Self-care Strategies") OR (MH "Self-concept") OR ("Self-regulation") OR ("Self-management") OR ("Self-careagency") OR ("Self-care demand") OR ("Self-care requisites") OR ("Self-monitoring") OR OR ("Self- medication") OR ("Symptom management") OR ("Patient autonomy*") OR (MH "Patient Compliance") OR (MH "Health behavior") OR (MH "Attitude to health") OR ("Attitude to illness") OR ("Patient attitude*") OR (MH "Choice behavior") OR (MH "Illness behavior") AND (intervention) AND ("insomnia" OR "hypersomnia" AND ("fatigue") AND ("weight loss" OR "weight gain" OR "appetite" AND ("anhedonia") AND ("irritable mood") AND ("self-esteem") AND ("guilt"). |
| Global ETD Search® | ("depressive disorder, major"[MeSH Terms] OR "major depression"[All Fields]) AND (MH "Self-care") OR(MH "Self-efficacy") OR ("Self-control") OR ("Self-care Behavior") OR ("Self-care Strategies") OR (MH "Self-concept") OR ("Self-regulation") OR ("Self-management") OR ("Self-careagency") OR ("Self-care demand") OR ("Self-care requisites") OR ("Self-monitoring") OR OR ("Self- medication") OR ("Symptom management") OR ("Patient autonomy*") OR (MH "Patient Compliance") OR (MH "Health behavior") OR (MH "Attitude to health") OR ("Attitude to illness") OR ("Patient attitude*") OR (MH "Choice behavior") OR (MH "Illness behavior") AND (intervention) AND ("insomnia" OR "hypersomnia" AND ("fatigue") AND ("weight loss" OR "weight gain" OR "appetite" AND ("anhedonia") AND ("irritable mood") AND ("self-esteem") AND ("guilt"). |
| EBSCO Open Dissertation® | ("depressive disorder, major"[MeSH Terms] OR "major depression"[All Fields]) AND (MH "Self-care") OR(MH "Self-efficacy") OR ("Self-control") OR ("Self-care Behavior") OR ("Self-care Strategies") OR (MH "Self-concept") OR ("Self-regulation") OR ("Self-management") OR ("Self-careagency") OR ("Self-care demand") OR ("Self-care requisites") OR ("Self-monitoring") OR OR ("Self- medication") OR ("Symptom management") OR ("Patient autonomy*") OR (MH "Patient Compliance") OR (MH "Health behavior") OR (MH "Attitude to health") OR ("Attitude to illness") OR ("Patient attitude*") OR (MH "Choice behavior") OR (MH "Illness behavior") AND (intervention) AND ("insomnia" OR "hypersomnia" AND ("fatigue") AND ("weight loss" OR "weight gain" OR "appetite" AND ("anhedonia") AND ("irritable mood") AND ("self-esteem") AND ("guilt"). |
| CAPES® theses and dissertations bank | ("depressive disorder, major"[MeSH Terms] OR "major depression"[All Fields]) AND (MH "Self-care") OR(MH "Self-efficacy") OR ("Self-control") OR ("Self-care Behavior") OR ("Self-care Strategies") OR (MH "Self-concept") OR ("Self-regulation") OR ("Self-management") OR ("Self-careagency") OR ("Self-care demand") OR ("Self-care requisites") OR ("Self-monitoring") OR OR ("Self- medication") OR ("Symptom management") OR ("Patient autonomy*") OR (MH "Patient Compliance") OR (MH "Health behavior") OR (MH "Attitude to health") OR ("Attitude to illness") OR ("Patient attitude*") OR (MH "Choice behavior") OR (MH "Illness behavior") AND (intervention) AND ("insomnia" OR "hypersomnia" AND ("fatigue") AND ("weight loss" OR "weight gain" OR "appetite" AND ("anhedonia") AND ("irritable mood") AND ("self-esteem") AND ("guilt"). |
| USP thesis and dissertation database | ("depressive disorder, major"[MeSH Terms] OR "major depression"[All Fields]) AND (MH "Self-care") OR(MH "Self-efficacy") OR ("Self-control") OR ("Self-care Behavior") OR ("Self-care Strategies") OR (MH "Self-concept") OR ("Self-regulation") OR ("Self-management") OR ("Self-careagency") OR ("Self-care demand") OR ("Self-care requisites") OR ("Self-monitoring") OR OR ("Self- medication") OR ("Symptom management") OR ("Patient autonomy*") OR (MH "Patient Compliance") OR (MH "Health behavior") OR (MH "Attitude to health") OR ("Attitude to illness") OR ("Patient attitude*") OR (MH "Choice behavior") OR (MH "Illness behavior") AND (intervention) AND ("insomnia" OR "hypersomnia" AND ("fatigue") AND ("weight loss" OR "weight gain" OR "appetite" AND ("anhedonia") AND ("irritable mood") AND ("self-esteem") AND ("guilt"). |
